# The Styryl Benzoic Acid Derivative DC10 Potentiates Radiotherapy by Targeting the xCT-Glutathione Axis

**DOI:** 10.3389/fonc.2022.786739

**Published:** 2022-02-07

**Authors:** Shahin Sarowar, Davide Cirillo, Pablo Játiva, Mette Hartmark Nilsen, Sarah-Muheha Anni Otragane, Jan Heggdal, Frode Selheim, Valentín Ceña, Hans-René Bjørsvik, Per Øyvind Enger

**Affiliations:** ^1^Oncomatrix Research Laboratory, Department of Biomedicine, University of Bergen, Bergen, Norway; ^2^Department of Chemistry, University of Bergen, Bergen, Norway; ^3^Unidad Asociada Neurodeath, Universidad de Castilla-La Mancha, Albacete, Spain; ^4^Centro de Investigación Biomédica en Red sobre Enfermedades Neurodegenerativas (CIBERNED), Instituto de Salud Carlos III (ISCIII), Madrid, Spain; ^5^Department of Medical Physics, Haukeland University Hospital, Bergen, Norway; ^6^The Proteomics Facility of the University of Bergen (PROBE), University of Bergen, Bergen, Norway; ^7^Department of Neurosurgery, Stavanger University Hospital, Stavanger, Norway

**Keywords:** SAR, oxidative stress, XCT, melanoma, radiosensitiser, glioma, mice

## Abstract

Metastatic tumors with moderate radiosensitivity account for most cancer-related deaths, highlighting the limitations of current radiotherapy regimens. The xCT-inhibitor sulfasalazine (SAS) sensitizes cancer cells to radiotherapy by blocking cystine uptake *via* the xCT membrane antiporter, and thereby glutathione (GSH) synthesis protecting against radiation-induced oxidative stress. The expression of xCT in multiple tumor types implies it as a target generic to cancer rather than confined to few subtypes. However, SAS has limited clinical potential as a radiosensitizer due to side effects and low bioavailability. Using SAS as a starting point, we previously developed synthetic xCT-inhibitors through scaffold hopping and structure optimization aided by structure-activity relationship analysis (SAR). Notably, the compound DC10 exhibited inhibition of GSH synthesis. In this study, we validated DC10 as a radiosensitizer in the xCT-expressing cancer cell lines A172, A375 and MCF7, and mice harboring melanoma xenografts. After DC10 treatment, we measured 14C-cystine uptake in the cancer cells using liquid scintillation counting, and intracellular GSH levels and reactive oxygen species (ROS) using luminescence assays. We performed immunoblotting of H2AX and ATM to assess DNA damage after treatment with DC10 and radiotherapy. We then assessed the effect of adding DC10 to radiation upon cancer cell colony formation. Blood samples from mice treated with DC10 underwent biochemical analysis to assess toxicity. Finally, mice with A375 melanomas in the flank, received DC10 and radiotherapy in combination, as monotherapies or no treatment. Notably, DC10 reduced cystine uptake and GSH synthesis and increased ROS levels in a dose-dependent manner. Furthermore, DC10 interacted synergistically with radiation to increase DNA damage and reduce tumor cell colony formation. Mice receiving DC10 were clinically unaffected, whereas blood samples analysis to assess bone marrow suppression, liver or kidney toxicity revealed no significant differences between treated mice and untreated controls. Importantly, DC10 potentiated the anti-tumor efficacy of radiation in mice with melanoma xenografts. We conclude that DC10 is well tolerated and acts as a radiosensitizer by inhibiting cystine uptake, leading to GSH depletion and increased oxidative stress. Our findings demonstrate the feasibility of using synthetic xCT-inhibitors to overcome radioresistance.

## Introduction

Ionizing radiation is a mainstay treatment for malignancies, and approximately 50% of cancer patients need it at some point (1). Moreover, the global burden of cancer is increasing and estimates of demands for radiotherapy suggest a further increase in the near future (2). Upon metastasis however, most cancer types are lethal, and the role of radiotherapy is limited to slowing disease progression and offering palliative relief. Although the idea of a radiosensitizer was conceptualized decades ago, this has not yet materialized. While immunotherapy and biological therapies directed towards oncogenic signalling pathways offer great hope, their efficacies are limited to cancer subtypes expressing their specific targets. Moreover, these drugs are often under patents, with spiralling costs that limit their availability and increase the strains on health economies worldwide. Thus, cancer radioresistance remains a major cause of human suffering and lost years, and currently account for roughly two-thirds of cancer-related deaths (3). As such, there is an unmet need for novel strategies to unleash the full potential of radiotherapy.

The therapeutic effect of radiation is mediated by ROS formation, leading to DNA damage and tumor cell death. ROS are also generated through cellular metabolism in the normal state where their levels are tightly regulated by ROS-scavenging antioxidants. Amongst these, glutathione (GSH) is abundantly present in mammalian cells and constitute a primary defence against oxidative stress. Moreover, its synthesis is homeostatically regulated, and can be increased in response to higher levels of ROS (4). Furthermore, the activities within various antioxidant systems in tumors are typically elevated to counteract increased ROS production due to higher metabolic rates and proliferative turnover (5). Notably, GSH levels are reportedly increased in several cancer types (6), and higher intracellular GSH levels have been linked to a poorer prognosis in patients with gastrointestinal cancers (7). In addition, total GSH levels as well as the ratio between reduced and oxidized GSH has been shown to increase in cancer cell lines following radiation (8). Collectively, these data imply major roles for GSH in mediating cancer radioresistance, suggesting that blocking GSH synthesis may enhance the anti-tumor efficacy of existing radiotherapy regimens.

Importantly, a rate-limiting step in GSH synthesis is the uptake of its precursor cystine *via* the membrane antiporter xCT in exchange for glutamate (9). xCT is expressed in multiple tumor types and has been linked to treatment resistance and poor prognosis (10, 11). Thus, the xCT antiporter provides an attractive target to combat radioresistance. Sulfasalazine (SAS) has been identified as a potent xCT-inhibitor (12), and experimental animal studies have demonstrated an added survival benefit when combining the drug with radiotherapy or cytotoxic agents (9, 13). Unfortunately, poor bioavailability of SAS due to intestinal degradation (14) and side effects (15, 16) have limited its use in heavily pre-treated cancer patients as a radiosensitizer. In recent years, several derivatives with a structural resemblance to SAS have been developed (17–19). However, these derivatives either share the diazo bond with the SAS molecule which undergoes cleavage in the gut by diazoreductases (18, 19), or its sulfapyridine moiety to which most side effects have been ascribed (17). Notably, they have not been biologically validated apart from their ability to inhibit cystine uptake and reduce metabolic activity in cancer cells.

With SAS as a starting point, we previously developed a novel molecular scaffold to increase metabolic stability (20). This was followed by additional structural modifications to reduce toxicity. Finally, optimization of potency against the xCT antiporter aided by structure-activity relationship analysis (SAR) approach resulted in several derivatives that reduced cystine uptake when tested in a panel of cancer cell lines expressing xCT (20). Amongst them, [(*E*)-5-(2-([1,1’-biphenyl]-4-yl) vinyl)-2-hydroxybenzoic acid] DC10 exhibited lower IC_50_ than SAS when tested in a panel of cancer cell lines (20). Furthermore, the diazo which undergoes enzymatic cleavage has been replaced with an olefinic bond (two carbon atoms linked by a double bond) in DC10 to avoid degradation by microbial azoreductases of the gut. Finally, the sulfonamide group, to which most side effects have been ascribed, has been replaced by an arene moiety in DC10. Given the promising results with DC10 in our initial screen, the compound was selected for further characterisation to assess its potential for clinical translation. Data pertaining to the validation of DC10 *in vitro* and *in vivo* is presented in this study.

## Materials and Methods

### Cell Culture

The cell lines used were A172 (glioma), A375 (melanoma) and MCF7 (breast adenocarcinoma), purchased from the American Type Culture Collection (ATCC, Manassas, VA, USA). Cell line identity was confirmed using DNA fingerprinting and tested negative for mycoplasma contamination using the MycoAlert kit (Lonza, Cat#: LT07-218). All cell culture work was performed aseptically in a sterile hood with laminar airflow. Cells were expanded in T75 flasks with Dulbecco’s modified eagle medium (DMEM, Thermo Fisher Scientific, Waltham, Massachusetts, United States) added with 10% fetal bovine serum, non-essential amino acids, 100 U/mL penicillin/streptomycin, and 400 μM L-glutamine (Lonza, Cologne, Germany) at 37°C in a humidified atmosphere of 5% v/v CO2.

### Drug Formulation

DC10 was stored in 100 mM aliquots at -20°C. DC10 along with a series of other styryl benzoic derivates were designed and synthesised by our group (20). DC10 was dissolved in dimethyl sulfoxide (DMSO), whereas physiological saline with equivalent concentration of DMSO served as controls. In the animal experiments, the compound was suspended and sonicated in physiological saline. To prevent degradation, new aliquots were used for each experiment.

### Measurements of Carbon-14 Labeled Cystine Uptake

Carbon-14 labeled cystine uptake was measured in the A172, A375 and MCF7 cell lines. A total of 50 000 cells per well were seeded in a 12 well plate and incubated for 24 hrs. After attachment, the cells were treated with 50 μM 100 μM and 150 μM doses of DC10 for 2 h prior to addition of L-[3,3′-14C]- cystine, (0.05 μCi/mL, PerkinElmer, Boston, MA, USA). After 20 h of incubation, the cells were rinsed twice with PBS, followed by the addition of RIPA buffer and scintillation cocktail. Radioactive cystine uptake was measured with a Tri Carb 3100 TR liquid scintillation analyzer (Packard Instrument Co., Inc., IL, USA). The assay was performed three times.

### GSH Assay

5000 cells from the A172, A375 and MCF7 cell lines were suspended in 100 µL that was loaded in each well of a white 96-well plate (Thermo Fisher Scientific, Waltham, Massachusetts, USA) and allowed to incubate for 24h. The medium was removed and replaced with an increasing concentration of DC10 from 25-1000 µM After 48 h of incubation, the media was removed and GSH-Glo™ reagent (Promega, Cat#: V6911) was added followed by 30 min incubation. 100 μL reconstituted luciferin detection reagent was added and incubated for another 15 min. The bioluminescence was quantified with the Victor3™ plate reader (PerkinElmer, Waltham, Massachusetts, USA) as counts per second with an integration time of one second. The averages of triplicate readings were exported to the GraphPad Prism software and expressed as fold change to DMSO controls. This procedure was repeated in separate experiments measuring GSH levels in the same cell lines without and with 10 mM NAC, untreated or treated with 50 μM 100 μM and 150 μM doses of DC10. All experiments were conducted three times.

### Immunoblotting

Cultured cells were treated with 50-150 μM of DC10 with or without radiation for 24 hours and lysed in Kinexus buffer, containing 20 mM MOPS, 5 mM EDTA, 0.5% Triton™ X-100, 2 mM EGTA, pH 7.2, as well as protease and phosphatase inhibitor cocktail tablets (Roche, Basel, Switzerland). Protein samples were purified, and the concentrations were measured using a BCA assay (Thermo Fisher Scientific, Waltham, Massachusetts, USA). 10-15 μg of protein sample was mixed with LDS sample-loading buffer and the reducing agent dithiothreitol and incubated at 70 °C for 10 min. Samples were run on a precast 4-12% gradient gels SDS-gel (Thermo Fisher Scientific, Waltham, Massachusetts, USA) followed by transfer to a nitrocellulose membrane. Following blocking for 1 h at room temperature, membranes were incubated with primary antibodies at 1:1000 dilution overnight at 4 °C. The antibodies used were: Rabbit anti- SLC7A11 Antibody (PA1-16775, Thermo Fisher Scientific), mouse anti-γH2AX (Ser139) (05-636, Merck millipore, Darmstadt, Germany), and rabbit anti-GAPDH (ab9485, Abcam, Cambridge, UK), rabbit anti- ATM (2873S), rabbit anti-phospho-ATM (Ser1981), rabbit anti- H2AX (7631S) (Cell signaling technology, Leiden, The Netherlands). After washing, the membranes were incubated with anti-rabbit and anti-mouse HRP secondary antibody according to the source of primary antibody at 1:10 000 dilution (Thermo Fisher Scientific) for 1.5 h at room temperature. Detection was done using Supersignal West Femto Maximum Sensitivity Substrate (Thermo Fisher Scientific, Waltham, Massachusetts, USA), and the membranes were developed using a Fuji LAS 3000 Imager (Fujifilm Medical Systems Inc.; Stamford, Connecticut, USA). Relative protein expression levels were normalized to GAPDH and quantified using Image J software (NIH; Bethesda, MD, USA). The western blotting experiments were conducted twice.

### Ionizing Radiation

Cancer cells were seeded in cell-culture plates and treated with increasing concentrations of DC10. After 24 h, 6 MV X-ray radiation was given as either 2 Gray (Gy) (monitor unit: MU1 185) in case of DNA damage assay, 4 Gy (MU1 371) in case of clonogenic assay, or 8 Gy (MU1 742) in case of *in vivo* experiment in a single dose in all experiments. The procedure was performed at room temperature at the Department of Oncology and Medical Physics, Haukeland University Hospital.

### ROS and Antioxidant Assays

Using Nunc™ MicroWell™ 96-well black clear-bottom Plates (Thermo Fisher Scientific, Waltham, Massachusetts, USA), 25 000 cells were seeded in each well and allowed to adhere overnight. The next day, ROS levels were detected using the DCFDA cellular ROS kit (ab113851, Abcam, Cambridge, UK) according to the manufacturer’s instructions. In short, the medium was removed, and the cells were washed with PBS and stained with 20 mM H2DCFDA for 45min at 37°C. After incubation, DCFDA was removed, and the cells were washed with PBS and medium (untreated cells) or medium with DC10 at 250, 500, and 1000 μM (treated cells) was added, with or without 10 mM of the antioxidant N-Acetyl Cysteine (NAC, Sigma-Aldrich, St. Louis, Missouri, USA). After 6 h of incubation, fluorescence was measured using a Victor3™ Plate Reader with excitation at 485 nm and emission at 535 nm. ROS levels were determined as relative to untreated controls and plotted in the GraphPad Prism software version 9.0 (GraphPad; La Jolla, CA, USA). The experiment was performed three times.

### Clonogenic Assay

Cells were seeded in triplicate from single cell suspensions in 6 well plates. The number of cells used was determined by the plating efficiency of the cell lines (1,000 cells/well in the cell lines used) (21). After attachment, the cells were treated with 50-150 μM of DC10 with or without NAC for 24 h, which was followed by 4 Gy radiation and the cells were kept undisturbed in the incubator for further observation. The cells multiply and form colonies usually after 10 to 14 days in controls. Each colony is composed of approximately 50 cells and visible by naked eyes. Then, the medium was removed gently, the wells were washed twice with 1X PBS solution, and the colonies were fixed with 300 μL of 4% v/v paraformaldehyde solution for 15 min followed by staining with 0.5% w/v crystal violet for 30 minutes. Excess crystal violet was washed with distilled water and plates allowed to dry. The colonies were counted manually, and the surviving fraction was calculated (21, 22) using Prism software version 9.0 (GraphPad; La Jolla, CA, USA). All experiments were performed 3times.

### Flow Cytometry

Flow cytometry was used to assess cell death analysis using Dead Cell Apoptosis Kits with Annexin V & Propidium Iodide (PI) (V13245, Thermo Fisher Scientific) according to the manufacturer’s instructions. In brief, 1 x 10^6^ A172, A375 and MCF cells were cultured in T25 culture flasks. After attachment, cells were treated with 50 µM and 100 µM of DC10 for 24h. Following washing with cold PBS, the cells were trypsinated, suspended in medium and spun down. The pellets were stained with 5 μl Annexin V Alexa Fluor 488 and 1 μl Propidium Iodide and incubated in dark at room temperature for 15 minutes. 400 μl Annexin V binding buffer was added to each sample and flow cytometry was performed on the BD Accuri C6 flow cytometer (Accuri Cytometers Ltd., Ann Arbor, Michigan, USA). Fluorescence was detected using filter FL1 for annexin V and FL3 for PI. For each sample, gated fluorescence properties of 20,000 cells were acquired. Further, the data were analyzed on FlowJo 10.8 (TreeStar, Ashland, OR, USA) software. Fluorescence signal overlapping of PI and FITC was compensated using unstained control, positive staining for Annexin V and PI. The experiment was performed twice.

### Animal Experiments

The animal experiments were performed in compliance with the guidelines for laboratory animals (23) and approved by the Norwegian Animal Research Authority (Oslo, Norway). NOD/SCID mice (NOD.CB17-PrkdcScid) weighing between 20-25 g were kept in the animal facility at the University of Bergen and provided a standard pellet diet and tap water ad libitum. They were kept in a pathogen-free environment at a constant temperature and humidity, and standard 12/12 h light and dark cycle. For the therapeutic study, animals were anesthetized with isoflurane gas (1.5% mixed with 50% air and 50% O_2_), and tumor implantation was conducted as previously described (24). A total of 5 x 10^5^ A375 cells were injected into the right flank of NOD/SCID mice. After ten days, animals with comparable tumor size were randomly assigned to the different treatment groups. DC10 was suspended in saline and 8 mg dose of DC10 was administered by oral gavage once daily on day 10 and 11. The 8 Gy radiation was given as a single session treatment, each procedure lasting less than two minutes for the animals. Dormicum was used as a sedative during the procedure (0.06mg Dormicum per gram mice subcutaneously). Animals were inspected daily, and tumor sizes were measured by a caliper every second or third day. All animals were asymptomatic for the duration of the experiment and were sacrificed at day 20 in a CO_2_ chamber, and the heart, lungs, liver, spleen, kidney, and biopsies of the small intestines were collected and fixed in 10% formaldehyde for 48 hours, embedded in paraffin. Tissue sections of the organs then underwent staining with Hematoxillin and Eosin (H/E). Control, radiotherapy, DC10, and DC10 in combination with radiation, each group comprised of 5 animals.

For the toxicity experiments, mice were fed 8 mg of DC10 suspended in 0.2 ml saline twice daily for three days and observed for 4 days. Blood samples were obtained upon sacrifice in CO_2_ chamber by cardiac puncture (25) using 3 mL syringes (Beckton Dickinson, Haryana, India) coupled with 23G gauge needles (Terumo Europe, Leuven, Belgium). Blood for hematological analysis was collected from the mice with a needle that was inserted upwards below the sternum (26). 250 μL of blood from each animal were added to tubes containing 1 mg potassium EDTA (Becton Dickinson, Plymouth, UK) for hematological analyses. The rest was collected in 1.5 mL tubes (Eppendorf, Hamburg, Germany), let coagulate for 30 minutes, and centrifuged at 2000 g for 10 minutes at room temperature. The supernatant containing the plasma fraction then underwent biochemical analysis at the Dept. of Clinical Chemistry at Haukeland University Hospital.

### Statistical Analysis

We used GraphPad Prism 9.0 (GraphPad Software Inc., La Jolla, CA, USA) for the statistical analysis. For cystine uptake, GSH assays, ROS assays and clonogenic assays, a two-way analysis of variance with Bonferroni’s multiple comparison test was performed. xCT expression levels in untreated and DC10-treated cells, and GSH levels in cells with and without NAC were analysed using the student’s t-test. Data were presented as means ± S.E.M, p ≦̸ 0.05 was considered significant. To obtain the IC_50_ dose for DC10-mediated depletion of GSH, the log of the measured values was calculated and plotted to generate a nonlinear regression fitted curve with variable slope. Median survival times for the treatment groups were compared using the log-rank (Mantel-Cox) test using the GraphPad Prism 9.0 statistical software. Tumor volumes were compared using the one-way ANOVA analysis.

## Results

### DC10 Inhibits Cystine Uptake, Leading to GSH Depletion and Increased ROS Levels

**Figure 1:**
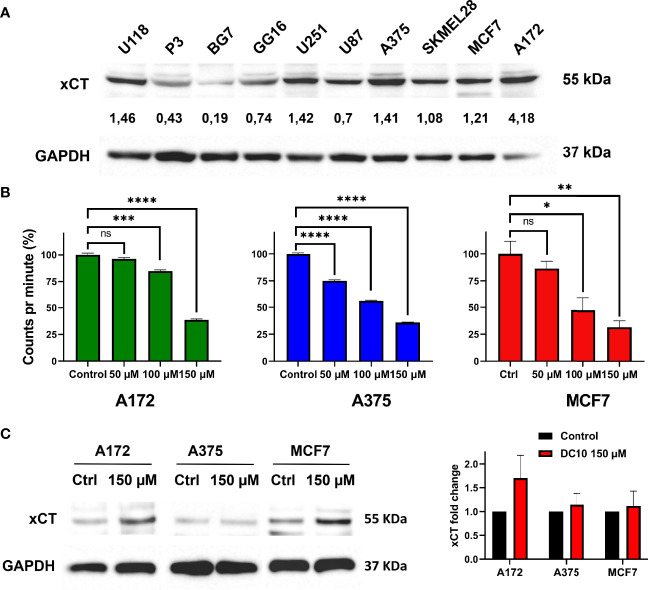
DC10 inhibits cystine uptake in xCT expressing cancer cell lines. **(A)** Western blot for the xCT antiporter in a panel of cancer cell lines. GAPDH was used as a loading control. **(B)** [^14^C] cystine uptake following treatment with 50 μM 100 μM and 150 μM doses of DC10 in the A172, A375 and MCF7 cell lines. **(C)** Western blots for xCT in the same cell lines in untreated and DC10 treated (left panel), with quantification of expression levels (right panel). *P < 0.05, **P < 0.01, ***P < 0.001, ****P < 0.0001. ns, not significant.

In order to assess the ability of DC10 to inhibit xCT- mediated cystine uptake required for GSH-synthesis, we screened a panel of cancer cell lines for their expression of xCT using western blotting (**Figure 1A**). Amongst these, we selected the A172 glioma cell line, A375 melanoma cell line and the MCF7 breast cancer cell line that all displayed robust expression of xCT for further studies. We then measured uptake of carbon-14 labelled cystine after treatment with increasing doses of DC10, comparing them to untreated controls (**Figure 1B**). Notably, DC10 blocked cystine uptake significantly in a dose-dependent manner in all 3 lines. To confirm that the reduced uptake was a result of specific xCT-inhibition and not downregulation of the xCT antiport, we performed western blotting for xCT in untreated and DC10-treated cells (**Figure 1C**). We did not detect significantly different expression of xCT after DC10 treatment in any of the cell lines compared to controls (p=0.23).

**Figure 2:**
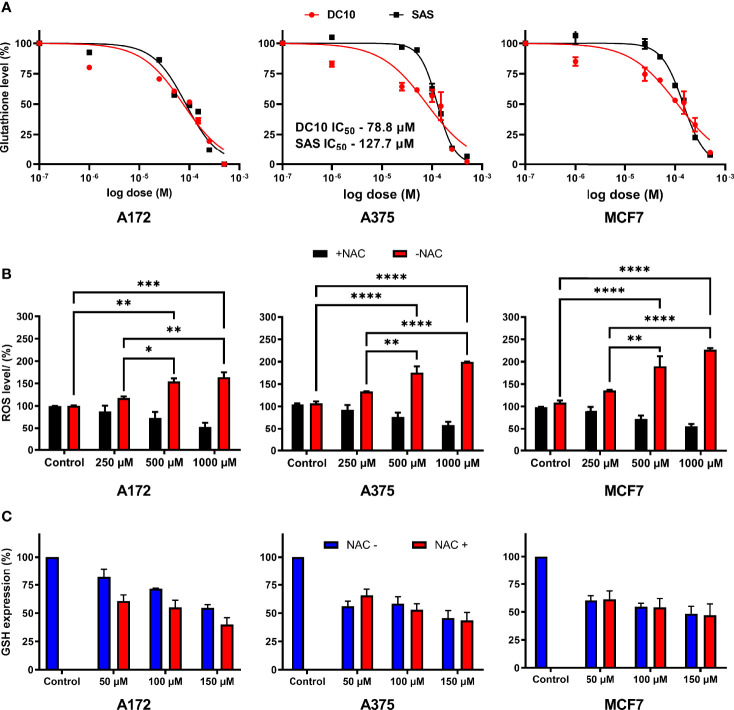
Dose-dependent effect of DC10 on GSH and ROS levels in cancer cell lines. **(A)** GSH levels in the A172, A375 and MCF7 cell lines in controls and after DC10 treatment with doses as indicated. **(B)** ROS levels after DC10 treatment with doses as indicated. *P < 0.05, **P < 0.01, ***P < 0.001, ****P < 0.0001. **(C)** GSH expression in DC10 treated cells in the presence or absence of NAC as indicated.

We next measured intracellular GSH levels in the A172, A375 and MCF7 cell cultures after 48 hours of incubation with increasing concentrations of DC10, as well as in untreated controls. Again, we observed a significant dose-dependent effect, with reduction of intracellular GSH levels in all the cell lines (**Figure 2A**), and IC_50_ doses were lower for DC10 than SAS in all the cell lines. Given the important role of GSH as a ROS scavenger, and as a cofactor in enzymatic degradation of peroxides, we next investigated how the reduction of intracellular GSH impacted on ROS levels. We measured ROS levels 6 hours after adding DC10, using the 2′,7′-Dichlorodihydrofluorescein diacetate probe (H2DCFDA), and observed a significant dose-dependent ROS increase in all the cell lines (**Figure 2B**). We next examined if increased ROS levels resulted from the loss of antioxidant properties exerted by GSH, by adding another antioxidant, N-Acetyl-L-Cysteine (NAC). Importantly, adding NAC reversed the effect of DC10 on ROS levels in all the cell lines (**Figure 2B**). To exclude the possibility that the effect of NAC was mediated by increased GSH synthesis, or by limiting the GSH-depleting action of DC10, we measured intracellular GSH levels in the presence and absence of NAC (**Figure 2C**). Importantly, we observed no significant difference in GSH levels between experiments in the presence or absence of NAC as DC10 also reduced GSH levels also in the presence of NAC (A172; p=0.97, A375; p=0.50, MCF7; p=0.45).

### DC10 and Radiation Synergistically Increases DNA Damage and Reduces Tumor Cell Colony Formation

**Figure 3:**
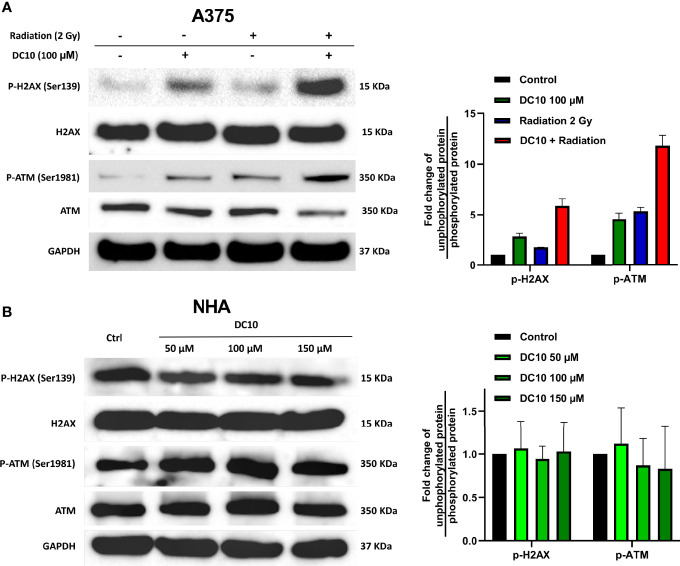
Synergistic effect of DC10 and radiation on DNA damage. Immunoblotting for unphosphorylated and phosphorylated forms of ATM and H2AX and GAPDH as a loading control in **(A)** A375 cells treated with DC10 and/or radiation and **(B)** NHA cells treated with DC10 as indicated. Ratios between phosphorylated and unphosphorylated forms expressed as fold change compared to untreated controls (right panels).

Since DC10 inhibited GSH synthesis that protects against oxidative stress, we wanted to investigate whether DC10 could sensitize cancer cells to increased ROS levels induced by radiotherapy. Hence, we conducted western blotting to assess expression of the DNA damage markers H2AX and ATM in the A375 cell line after combined treatment with DC10 and radiation (**Figure 3A**, left panel). Although both DC10 and radiation produced DNA damage as monotherapies, there was a marked increase when they were co-administered (**Figure 3A**, both panels). Since DC10 alone induced DNA damage in the cancer cell lines, we subsequently treated normal human astrocytes (NHA) with 50 μM 100 μM and 150 μM doses of DC10 to investigate if it was also genotoxic to healthy cells (**Figure 3B**). We did not observe increased DNA damage after DC10-treatment at any dose compared to untreated controls.

**Figure 4:**
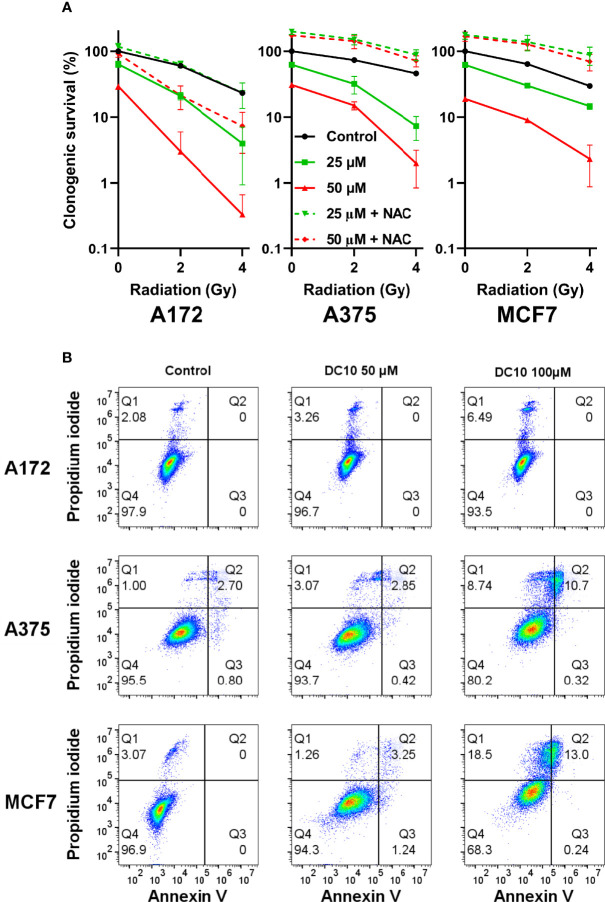
Synergistic effect of DC10 and radiation on tumor cell colony formation **(A)** Clonogenic survival in cancer cell lines after 2 and 4 Gy radiation and treatment with 25 µM and 50 µM doses of DC10 in the presence or absence of NAC as indicated. **(B)** Scatter plot of untreated and DC10 treated cells stained for propidium iodide and annexin V.

We then examined the effect of adding DC10 to radiotherapy on cancer cell colony formation in clonogenic assays using the A172, A375, and MCF7 cell lines. Thus, cell cultures receiving 2 Gy or 4 Gy of irradiation were incubated with 25 µM and 50 µM of DC10 and colonies were assessed after 10 to 14 days. Whereas both DC10 and radiation reduced colony formation in a dose-dependent manner, the combined effect was larger than the sum of each treatment in all the cell lines, indicating that DC10 interacted synergistically with radiation to increase DNA damage and anti-tumor efficacy (**Figure 4A**). Again, we observed that NAC consistently reversed the effect of DC10 and even increased colony formation to higher levels than observed in untreated controls for some of the groups. Since DC10 alone reduced colony formation in all the cancer cell lines, we conducted additional flow cytometry to assess necrotic and pre-apoptotic cells after 24 hours using propidium iodide and Annexin V staining (**Figure 4B**). These studies confirmed that DC10 alone triggered dose-dependent, but varying levels of cancer cell death already at an early time point.

### DC10 Is Well Tolerated in Mice and Sensitizes Melanoma Xenografts to Radiotherapy

**Figure 5:**
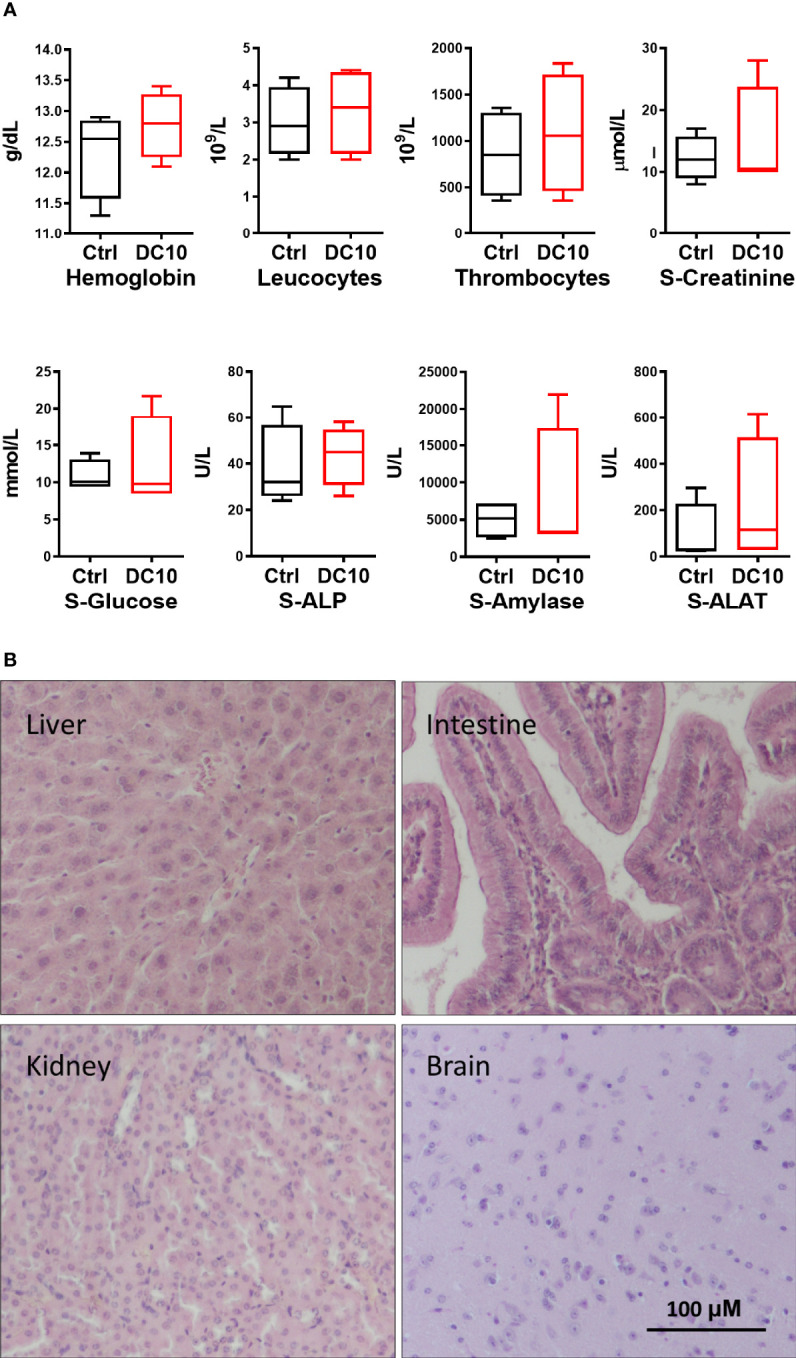
Toxicology screening of DC10 *in vivo*. **(A)** Blood cells and biochemical analysis of samples from animals receiving DC10 (red) or controls receiving vehicle (black). S-ALP, Serum alkaline phosphatase; S-ALAT, Alanine transaminase. **(B)** H/E-stained sections of organs harvested from mice 10 days post DC10-treatment. 40x, Scale bar: 100 µM.

In order to assess whether therapeutic use of DC10 as a radiosensitizer was feasible, we conducted a preliminary toxicology screening in NOD-SCID mice, administering DC10 at equimolar doses twice the doses of SAS previously used in animal experiments. The animals receiving DC10 were clinically not affected and had stable weight during 14 days of observation. Furthermore, hematological and biochemical analysis of blood sample from the mice 4 days after completed DC10 treatment, revealed no significant difference between treated animals and controls (**Figure 5A**). We also performed histopathological examination of organs from mice 10 days after receiving DC10 and from untreated controls. We did not detect any signs of toxicity in the treated animals and confirmed normal histoarchitecture identical to untreated mice (**Figure 5B**).

**Figure 6:**
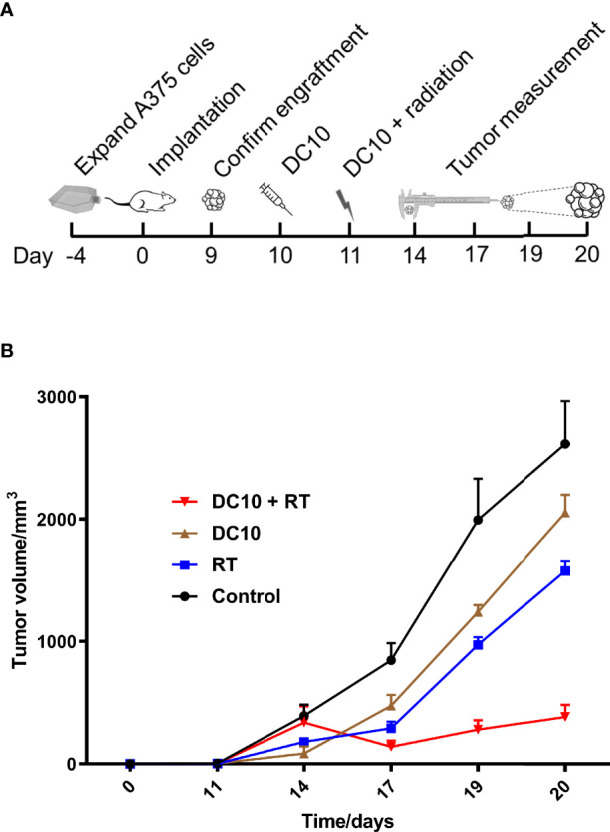
DC10 interacts synergistically with radiotherapy to suppress growth of A375 melanoma xenografts in NOD-SCID mice. **(A)** Overview with timeline for the *in vivo* experiment. **(B)** Growth curves of A375 flank tumors for the different treatment groups, Ctrl: Control (n=6), DC10: DC10 treatment (n=5), RT: Radiotherapy (n=6) and DC10 + RT: Combination treatment (n=6).

In the next experiment, NOD-SCID mice with A375 cells implanted in the flank, and upon engraftment of the tumor xenografts mice were randomly assigned on day 10 into the following treatment groups: 1) DC10, 2) 4 Gy radiation to the tumor area, 3) DC10 and 4 Gy radiation, and 5) Controls receiving no treatment (**Figure 6A**). Radiotherapy was administered on day 11 after implantation. We subsequently observed a rapid increase in tumor volume in the untreated control group (**Figure 6B**). Compared to the controls, tumors treated with DC10 grew significantly slower (p=0.05) as did tumors receiving radiation (p=0.01). However, the most striking response was observed in the group receiving both DC10 and radiation in which tumors initially shrunk from the time of treatment to the next measurement 3 days later. Although these tumors resumed growth it was significantly slower than in the other treatment groups (p=0.0004). Due to the tumor size in the control group, the experiment was terminated on day 20 post-implantation.

## Discussion

In summary, the present data demonstrate that: 1) DC10 increases oxidative stress by blocking cystine uptake needed for GSH synthesis, 2) DC10 is well tolerated in mice when administered at therapeutic doses, and 3) DC10 interacts synergistically with radiation to inhibit tumor growth.

DC10 was developed with the xCT-inhibitor SAS molecule as a starting point and inhibit cystine-uptake dose-dependently similar to SAS. Western blotting did not demonstrate any change in expression levels of xCT in the absence or presence of DC10, confirming that the reduced uptake is due to inhibition and not downregulation of the antiporter. The resulting reduction of intracellular GSH was accompanied by increased fluorescence signal as measured by the dichlorofluorescin ROS assay, that was reversed by adding the antioxidant NAC. The interpretation of these findings is not without reservations since several studies show that other factors than elevated ROS can increase signal intensity such as lowering of GSH levels (27). However, DC10 treatment alone also increased DNA damage, reduced clonogenicity and triggered cancer cell death which is consistent with a ROS increase. That cancer cells are vulnerable to GSH lowering alone likely reflect their strong dependency on protection against oxidative stress, due to the increased ROS production that accompanies their high metabolic rates and proliferative turnover. Notably, we did not observe increased DNA damage in non-transformed astrocytes after DC10 treatment.

The cell lines we used represent different cancer types, reflecting the fact that the xCT antiporter is broadly expressed in tumors of all organ systems (28). Thus, the xCT/GSH-axis has emerged as a target that is generic to cancer rather than restricted to certain cancer subtypes. Accordingly, this axis has gained interest and multiple non-FDA approved xCT inhibitors, also without a structural resemblance to SAS, have been developed or identified through drug screens (29, 30). Conversely, the xCT-inhibitor Sorafenib is FDA approved but as a multi-kinase inhibitor mainly for kidney and liver cancer. However, severe toxicity was observed when it was combined with radiotherapy, which likely resulted from its multiple targets and actions (31). DC10 was previously developed through a series of structural modifications of the SAS molecule to obtain a compound with fine-tuned properties for clinical use as a radiosensitiser. SAS itself was designed as a prodrug and is mostly metabolized by gut bacteria in the intestines through cleavage of its diazo bond into sulfapyridine and 5-aminosalicylic acid. Importantly, neither of these metabolites display activity against the xCT antiporter (12), and SAS has therefore been administered intraperitoneally to avoid intestinal degradation when used as an xCT inhibitor in animal experiments (9, 32). In order to avoid cleavage by bacterial azo reductases we replaced the diazo bond with an olefinic bond. This allowed us to administer DC10 by oral gavage instead of intraperitoneally which markedly reduced tumor growth in combination with radiotherapy and is consistent with improved metabolic stability of DC10 compared to SAS. However, extensive pharmacological profiling is needed to precisely estimate systemic availability and biological half-life prior to clinical use of DC10.

A particular challenge with using SAS in heavily pre-treated cancer patients is posed by its side effects that overlap with those of cytotoxic agents commonly used to treat malignancies. Thus, the sulfapyridine moiety of SAS was removed in DC10 as it is associated with bone marrow suppression and adverse reactions in multiple organ systems. Notably, SAS-related toxicity including cytopenia led to early termination of one clinical trial enrolling brain tumor patients (15) and discontinuation of the drug in the majority of patients in another study (16). Conversely, DC10 was well tolerated in our study and treated mice did not display signs of hematologic side effects. It should be emphasized however, that SAS was administered for prolonged periods in both the patient trials, whereas we used single session radiotherapy necessitating only a two days’ course of treatment with DC10. Thus, a more extensive toxicology screening as well as longer duration of treatment, will be required prior to testing in patients.

## Data Availability Statement

The raw data supporting the conclusions of this article will be made available by the authors, without undue reservation.

## Ethics Statement

The animal study was reviewed and approved by Norwegian Animal Research Authority.

## Author Contributions

Conceptualization, PE and H-RB. Methodology, SS, DC, PJ, MN, S-MO, JH, FS, H-RB, and PE. Validation, SS, DC, and PE. Formal analysis, SS and PE. Investigation, SS, DC, PJ, S-MO, FS, and PE. Resources, VC, H-RB, and PE. Data curation, SS and PE. Writing—original draft preparation, SS. Writing—review and editing, PJ, VC, PE, and H-RB. Supervision, PE and H-RB. Project administration, PE and H-RB. Funding acquisition, PE and H-RB. All authors have read and agreed to the published version of the manuscript.

## Funding

This work is supported in part by the Norwegian research council grant nr. 295740. SS acknowledges his PhD grant funded by Department of Biomedicine at the University of Bergen. DC acknowledges his ESR grant through the PET3D project that was funded by the European Commission under the H2020 – MSCA-ITN-2015 program grant agreement no 675417.

## Conflict of Interest

SS, DC, H-RB, and PE have ownership in the Patent PCT/GB2020/052335 under which the compound DC10 is protected.

The remaining authors declare that the research was conducted in the absence of any commercial or financial relationships that could be construed as a potential conflict of interest.

## Publisher’s Note

All claims expressed in this article are solely those of the authors and do not necessarily represent those of their affiliated organizations, or those of the publisher, the editors and the reviewers. Any product that may be evaluated in this article, or claim that may be made by its manufacturer, is not guaranteed or endorsed by the publisher.
